# Protective effects of quercetin against glyphosate-induced nephrotoxicity in rats: role of oxidative stress, inflammatory response, and apoptotic pathways

**DOI:** 10.3389/fvets.2025.1624763

**Published:** 2025-09-10

**Authors:** Ashraf Albrakati

**Affiliations:** Department of Anatomy, College of Medicine, Taif University, Taif, Saudi Arabia

**Keywords:** glyphosate, quercetin, nephrotoxicity, oxidative stress, inflammation, apoptosis, antioxidant

## Abstract

**Background:**

Glyphosate, the most widely used herbicide globally, accumulates in renal tissue causing kidney damage through incompletely understood mechanisms. This study evaluated quercetin’s nephroprotective effect against glyphosate-induced kidney injury in rats.

**Methods:**

Five groups of male Wistar rats (*n* = 10 each) received daily treatments for 21 days: control, glyphosate (25 mg/kg), quercetin (50 mg/kg), and quercetin+glyphosate at low (25 mg/kg) or high (50 mg/kg) doses. All treatments were administered by oral gavage for 21 days. Renal parameters, oxidative stress markers, inflammatory mediators, and apoptotic indicators were assessed using spectrophotometric assays, ELISA, qRT-PCR, and histology.

**Results:**

Glyphosate impaired renal function, increased kidney weight, and elevated kidney injury molecule-1 (KIM-1) levels. It suppressed antioxidant enzymes (CAT, SOD, GPX) and downregulated their mRNA expression (*Cat*, *Sod2*, and *Gpx-1*, respectively), while depleting GSH and increasing oxidative markers (MDA, NO). Notably, glyphosate reduced Nrf2 protein and *Nfe2l2* gene expression, disrupting this master regulator of antioxidant responses, with concurrent *Hmox-1* downregulation. Glyphosate upregulated pro-inflammatory cytokines (TNF-*α*, IL-1β, IL-6), increased TLR-4 and *NOS2* expression, activated mitochondrial apoptosis by increasing pro-apoptotic proteins (BAX, CYTOCHROME C, and CASPASE-3) while decreasing anti-apoptotic BCL-2 protein levels, with corresponding changes in gene expression. Consistent with protein findings, *Bcl-2* gene expression was significantly downregulated, further confirming the shift toward pro-apoptotic signaling. Quercetin dose-dependently attenuated these alterations, with high-dose providing superior protection compared to low-dose by restoring gene expression and enzyme activities. Histopathological examination confirmed quercetin mitigated glyphosate-induced tubular degeneration and glomerular atrophy.

**Conclusion:**

Quercetin protects against glyphosate nephrotoxicity through antioxidative, anti-inflammatory, and anti-apoptotic mechanisms, suggesting therapeutic potential against herbicide-induced kidney injury.

## 1 Introduction

Nephrotoxicity represents a significant global health concern characterized by deteriorating renal function, resulting in metabolic waste accumulation, electrolyte imbalances, and increased risk of renal failure (1). Multiple factors contribute to kidney injury, including ischemia–reperfusion injury, nephrotoxic medications, sepsis, and environmental toxicants (2). Among environmental xenobiotics, glyphosate [N-(phosphonomethyl) glycine], the world’s most widely used herbicide, has emerged as a concerning potential nephrotoxic agent with increasing clinical relevance (3).

Glyphosate is extensively used in agriculture for weed control in crops like soybean, corn, and cotton, as well as in non-agricultural settings for vegetation management (4). In many ecosystems, substances like glyphosate accumulate through the food chain, ultimately affecting human health through chronic low-dose exposure (5).

Given its preferential accumulation in the kidneys more so than in the spleen, liver, or neural tissue (5), glyphosate raises particular concern for nephrotoxicity, especially during sub-acute (6) and sub-chronic exposure (7) scenarios.

The precise mechanisms of glyphosate-induced nephrotoxicity remain incompletely understood. However, emerging evidence suggests oxidative stress plays a pivotal role in the pathogenesis of glyphosate-induced renal injury (8). Prolonged exposure suppresses endogenous antioxidant defense systems [catalase (CAT), superoxide dismutase (SOD), and glutathione peroxidase (GPX)] while enhancing reactive oxygen species (ROS) production. This persistent oxidative imbalance leads to lipid peroxidation, protein oxidation, and DNA damage, culminating in renal cellular dysfunction and death (9).

Additionally, glyphosate exposure activates inflammatory pathways, notably through Toll-like receptor 4 (TLR-4) signaling, resulting in pro-inflammatory cytokine production [tumor necrosis factor-*α* (TNF-α), interleukin-1β (IL-1β), interleukin-6 (IL-6)] (10). This inflammatory cascade exacerbates renal injury through progressive leukocyte infiltration and tubular damage [15]. The mitochondrial apoptotic pathway, characterized by altered Bcl-2-associated X protein (BAX)/ B-cell lymphoma 2 (BCL-2) ratio, CYTOCHROME C release, and CASPASE-3 activation, has been implicated in glyphosate-induced renal cell death during continuous exposure (11).

Given widespread glyphosate exposure and limited treatments for chemical-induced nephrotoxicity, effective interventions were urgently needed. Natural antioxidants have gained significant attention for mitigating nephrotoxicity by modulating oxidative stress, inflammation, and apoptotic signaling (12). Quercetin (3,3′,4′,5,7-pentahydroxyflavone), a plant-derived flavonoid, demonstrates potent antioxidant, anti-inflammatory, and anti-apoptotic properties (13).

Quercetin’s protective effects stem from free radical scavenging and enhancement of endogenous antioxidant defenses, while also regulating inflammatory mediators and inhibiting inflammatory pathways (14). Previous studies have shown quercetin has protective effects against liver injury (15), kidney injury (16), and other model diseases such arteriosclerosis (14), but its potential against glyphosate-induced nephrotoxicity and underlying molecular mechanisms remain largely unexplored.

Recent evidence suggests the interaction between oxidative stress and inflammatory pathways represents a critical axis in xenobiotic-induced kidney injury (17), creating a self-amplifying cycle leading to mitochondrial dysfunction, apoptosis, and renal impairment (18). This study aimed to investigate quercetin’s nephroprotective effects against glyphosate-induced kidney injury in rats exposed for 3 weeks, hypothesizing that quercetin would attenuate renal damage by modulating oxidative stress, inflammatory responses, and apoptotic pathways during sub-acute exposure.

## 2 Materials and methods

### 2.1 Experimental animals

Fifty male Wistar rats (150–200 g) were purchased from King Fahd Medical Research Center (KFMRC), King Abdulaziz University, Saudi Arabia. Before the start of the experiment, animals were acclimatized for 1 week under standard conditions. Rats were housed in plastic cages at room temperature (22–25°C) with a 12-h light/dark cycle, and had free access to standard laboratory diet and water.

### 2.2 Chemicals

Glyphosate (CAS No. 1071-83-6; Cat: 337757) and Quercetin (CAS No. 117–39-5; Cat: Q4951) were purchased from Sigma-Aldrich Co. LLC., St. Louis, MO, USA and freshly prepared in distilled water prior to administration.

### 2.3 Animal grouping and treatment

A total of 50 male Wistar rats were randomly divided into five groups (n = 10 per group). All treatments were administered once daily via oral gavage for 21 consecutive days. The groupings were as follows:

Group 1 (Control group, Control): Rats received 0.9% physiological saline intraperitoneally as a vehicle control.

Group 2 (Glyphosate group, Glyphosate): Rats received glyphosate at a dose of 25 mg/kg/day (19) to induce sub-acute nephrotoxicity.

Group 3 (Quercetin group, Quercetin): Rats received quercetin alone (50 mg/kg/day) (20) to assess its impact on normal renal function and exclude any intrinsic toxicity of the high dose.

Group 4 (Quercetin low-dose + Glyphosate group, Que-25 + Glyp): Rats received quercetin (25 mg/kg/day) (21), administered 1 h prior to glyphosate injection to evaluate the protective effect of a low dose.

Group 5 (Quercetin high-dose + Glyphosate group, Que-50 + Glyp): Rats received quercetin (50 mg/kg/day), administered 1 h prior to glyphosate injection to assess the dose-dependent protective effects against glyphosate-induced renal damage.

Sample size was determined based on previous similar toxicological studies evaluating nephroprotective agents in rats, with consideration of anticipated effect size, variability, and statistical power. A group size of 10 animals per group was selected to ensure sufficient power (80%) to detect significant differences with a confidence level of 95% and considering potential data loss.

### 2.4 Anesthesia and sample collection

On day 22, animals were anesthetized with thiopental sodium (100 mg/kg, intraperitoneally). Adequate anesthesia was confirmed by the absence of the pedal withdrawal reflex. Blood samples were collected immediately via cardiac puncture using sterile 5 mL syringes with 21-gage needles under aseptic conditions (as it enables sufficient terminal blood collection for biochemical and molecular analyses). The animals were then euthanized by cervical decapitation. Kidneys were excised, rinsed with ice-cold saline, and processed for histological, biochemical, and molecular analyses. Blood samples were allowed to clot at room temperature for 30 min and centrifuged at 3000 rpm for 10 min. The obtained serum was separated and stored at −80°C until biochemical analysis.

### 2.5 Serum renal function tests

Renal function was assessed by measuring serum creatinine, urea, and uric acid, using commercially available diagnostic kit (Randox Laboratories Ltd., Crumlin, County Antrim, UK). All assays were performed according to the manufacturers’ protocols.

### 2.6 Determination of kidney weight

The relative kidney weight was calculated according to the method described by Almeer et al. (22). To ensure consistency across all samples, the left kidney was selected for weight measurement. The right kidney was preserved for histopathological and molecular analyses requiring intact tissue integrity.

### 2.7 Relative kidney weight

Relative kidney weight was calculated using the formula: (left kidney weight / body weight) × 100.

### 2.8 Renal injury biomarkers

Kidney Injury Molecule-1 (KIM-1), a highly sensitive early biomarker of proximal tubular damage, was measured in kidney tissue homogenates using ELISA kit (Elabscience Biotechnology Inc., Houston, TX, USA) according to the manufacturer’s instructions.

### 2.9 Redox status determination

#### 2.9.1 Oxidative stress markers

Oxidative stress markers were assessed in kidney tissue homogenates to evaluate the extent of cellular damage induced by glyphosate exposure. Malondialdehyde (MDA) levels were determined the procedure established by Ohkawa et al. (23). Reduced glutathione (GSH) content was determined using the method outlined by Ellman (24). Nitric oxide (NO) content in renal samples was measured by Griess reagent (25).

#### 2.9.2 Antioxidant enzyme activities

To assess the antioxidant defense system, several key enzymatic activities were measured. SOD activity was evaluated using the technique described by Nishikimi et al. (26). CAT activity was measured using the method of Lück (27). GPX activity was assessed using the technique described by Paglia and Valentine (28). The level of glutathione reductase (GR,) was assessed using the technique described by Moron et al. (29). Nuclear factor erythroid 2-related factor 2 (Nrf2) levels were quantified using ELISA kit (Elabscience Biotechnology Inc., Houston, TX, USA) according to the manufacturer’s instructions.

### 2.10 Inflammatory cytokines assessment

Levels of tumor necrosis factor-alpha in kidney tissue homogenates were quantified using commercial ELISA kits (TNF-*α*), IL-1β, and IL-6 (Elabscience Biotechnology Inc., Houston, TX, USA), according to the manufacturer’s instructions.

### 2.11 Apoptotic markers

Apoptotic proteins in kidney tissue were quantified using commercial ELISA kits. BCL-2 and BAX levels were measured with kits from BioVision Inc., Milpitas, CA, USA and expressed as ng mg−1 protein. CASPASE-3 activity was determined with a colorimetric assay kit from BioVision Inc., Milpitas, CA, USA, while CYTOCHROME C levels were assessed using an ELISA kit from Elabscience Biotechnology Inc., Houston, TX, USA (expressed as nmol mg−1 protein) to evaluate mitochondrial integrity. All assays were performed in accordance with the respective manufacturers’ protocols.

### 2.12 Gene expression analysis

Total RNA was extracted from kidney tissue samples using TRIzol reagent (Invitrogen, Carlsbad, CA, USA) according to the manufacturer’s instructions. RNA purity and concentration were determined spectrophotometrically using NanoDrop (Thermo Fisher Scientific, Waltham, MA, USA) by measuring absorbance at 260/280 nm. Complementary DNA (cDNA) was synthesized from 1 μg of total RNA using RevertAid™ H Minus Reverse Transcriptase (30) following the manufacturer’s protocol. Quantitative real-time PCR (qRT-PCR) was performed using QuantiFast SYBR Green PCR kit (Qiagen, Hilden, Germany) on an Applied Biosystems 7,500 system (Thermo Fisher Scientific, CA, USA). The PCR cycling conditions included initial denaturation at 95°C for 10 min, followed by 40 cycles of denaturation at 95°C for 15 s and annealing/extension at 60°C for 60s. All reactions were conducted in triplicate, and the relative gene expression levels were calculated using the 2^–ΔΔCt method with β-actin (Actb) as the internal control (30). Target genes analyzed included antioxidant response markers (Nfe2l2, Hmox-1, Sod2, Cat, GPx-1), inflammatory markers (TLR-4, TNF-α, IL-6, IL-1β), antioxidative stress markers (nitric oxide synthase NOS2), and apoptotic markers (Bax, Bcl-2, Caspase-3, Cycs). Primer sequences for all genes were listed in Table 1.

**Table 1:** List of primer sequences of the genes analyzed by qRT-PCR.

Name	Accession number	Sense primer (5′ → 3′)	Antisense primer (5′ → 3′)
*Nfe2l2*	NM_031789.2	CAGCATGATGGACTTGGAATTG	GCAAGCGACTCATGGTCATC
*Hmox-1*	NM_012580.2	TTAAGCTGGTGATGGCCTCC	GTGGGGCATAGACTGGGTTC
*Sod2*	NM_017051.3	AGCTGCACCACAGCAAGCAC	TCCACCACCCTTAGGGCTCA
*Cat*	NM_012520.2	TCCGGGATCTTTTTAACGCCATTG	TCGAGCACGGTAGGGACAGTTCAC
*Gpx-1*	NM_030826.2	CGGTTTCCCGTGCAATCAGT	ACACCGGGGACCAAATGATG
*NOS2*	NM_012611.3	GGTGAGGGGACTGGACTTTTAG	TTGTTGGGCTGGGAATAGCA
*Tnfα*	NM_013693.3	AGAGGCACTCCCCCAAAAGA	CGATCACCCCGAAGTTCAGT
*IL-1β*	NM_008361.4	TGCCACCTTTTGACAGTGATG	TTCTTGTGACCCTGAGCGAC
*Bax*	NM_007527.3	CTGAGCTGACCTTGGAGC	GACTCCAGCCACAAAGATG
*Bcl-2*	NM_009741.5	GACAGAAGATCATGCCGTCC	GGTACCAATGGCACTTCAAG
*Caspase-3*	NM_001284409.1	GAGCTTGGAACGGTACGCTA	CCGTACCAGAGCGAGATGAC
*Cycs*	NM_012839.2	CTTGGGCTAGAGAGCGGGA	TGAAGCACGGGTGAGTCTTC
*TLR-4*	NM_019178.2	TGGATACGTTTCCTTATAAG	GAAATGGAGGCACCCCTTC
*TNF-α*	NM_013693.2	CCCTCACACTCAGATCATCTTCT	GCTACGACGTGGGCTACAG
*IL-6*	NM_012589.2	AGTTGCCTTCTTGGGACTGA	TCCACGATTTCCCAGAGAAC
*Actb*	NM_007393.5	CTCTAGACTTCGAGCAGGAGATGG	ATGCCACAGGATTCCATACCCAAGA

Gene: Nfe2l2 (nuclear factor erythroid 2-related factor 2), Hmox1 (heme oxygenase 1), Sod2 (superoxide dismutase 2), Cat (catalase), Gpx1 (glutathione peroxidase 1), Nos2 (nitric oxide synthase 2), Tnf-α (tumor necrosis factor alpha), IL-1 β (interleukin 1 beta), Bax (BCL2-associated X protein), Bcl2 (B-cell lymphoma 2), Caspase-3 (caspase 3), Cycs (cytochrome c, somatic), TLR-4 (toll-like receptor 4), IL-6 (interleukin 6), Actb (beta-actin).

### 2.13 Histopathological examination

Kidney samples were fixed in 10% formalin for 24 h, dehydrated through graded ethanol (70, 80, 90, and 100% for 5 min each), cleared in xylene, embedded in paraffin wax, sectioned at 5 μm, and stained with hematoxylin and eosin (H&E) (31). The stained sections were evaluated under a light microscope (Nikon Eclipse E200) to assess pathological changes.

Histopathological evaluation: Renal tissue sections were scored semi-quantitatively in a blinded manner using the Endothelial–Glomerular–Tubular–Interstitial (EGTI) scoring system, as previously described by Toprak et al. (32). Lesions were graded on a 0–3 scale for each component (0 = none, 1 = mild, 2 = moderate, 3 = severe, 4 = very severe). Three non-overlapping fields per section were examined at 400 × magnification by a blinded pathologist. Data were presented as mean ± standard deviation (SD). Statistical significance was assessed using one-way ANOVA followed by Tukey’s *post hoc* test for multiple comparisons.

### 2.14 Statistical analysis

All data were presented as mean ± SD. Statistical comparisons among experimental groups were conducted using one-way analysis of variance (ANOVA), followed by Tukey’s post hoc test to determine pairwise differences. Statistical analyses were performed using SPSS software (version 16.0). A *p*-value < 0.05 was considered statistically significant. All biochemical assays were conducted in triplicates to ensure accuracy and reproducibility. Statistical notation: ^#^ indicates significant difference vs. control; ^$^ indicates significant difference vs. glyphosate (*p* < 0.05).

## 3 Results

### 3.1 Kidney weight and relative kidney weight assessment

**Figure 1:**
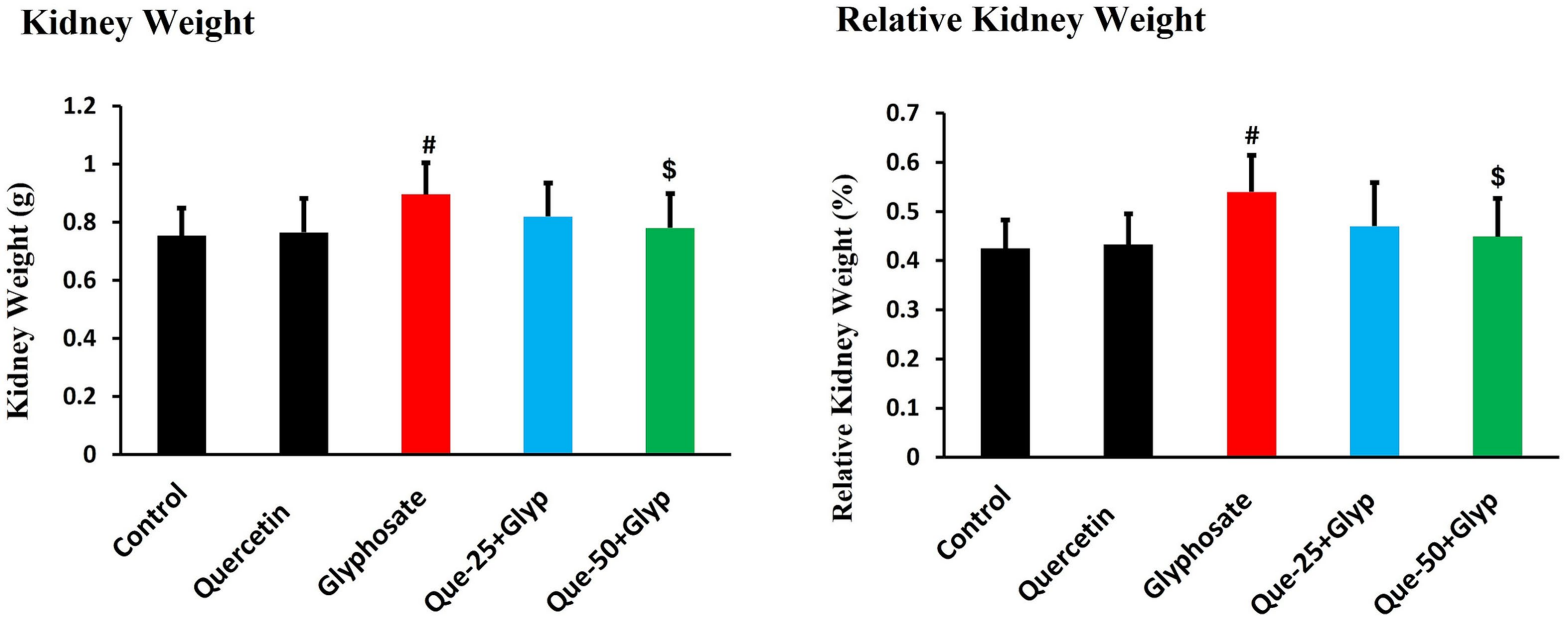
Effects of glyphosate and quercetin on kidney weight and relative kidney weight. Rats were administered with glyphosate and/or quercetin at low dose or high dose for 21 days. Results were presented as mean± SD (*n* = 10). Statistical significance: ^#^ indicates significant difference compared to the control group (*p* < 0.05); ^$^ indicates significant difference compared to the glyphosate-treated group (*p* < 0.05).

Morphometric analysis revealed significant alterations in kidney weight parameters across experimental groups. Glyphosate administration significantly increased kidney weight and relative kidney weight compared to controls (*p* < 0.05), indicating renal hypertrophy possibly due to inflammatory processes or cellular edema. Quercetin treatment dose-dependently attenuated these morphological alterations, with high-dose quercetin normalizing values to near-control levels, while low-dose treatment showed moderate improvement (Figure 1). This progressive normalization represents initial macroscopic evidence for quercetin’s nephroprotective properties. No statistically significant differences were observed between the initial and final body weights within each group, including the control and glyphosate-treated groups (*p* > 0.05, Table 2).

**Table 2:** Mean ± SD of initial and final body weights of experimental animals in different treatment groups.

Group	Initial body weight (g)	Final body weight (g)
Control	170.4 ± 13.75	178.8 ± 13.12
Quercetin	168.7 ± 12.86	176.2 ± 12.23
Glyphosate	159.6 ± 13.30	157.7 ± 13.40
Que-25 + Glyp	174.3 ± 12.35	178.3 ± 12.95
Que-50 + Glyp	171.0 ± 12.32	176.60 ± 12.25

Data were presented as mean ± standard deviation (SD). Statistical analysis was performed using paired *t*-test to compare the initial and final body weights within each group. No statistically significant differences were observed (*p* > 0.05).

### 3.2 Renal function and injury biomarkers

**Figure 2:**
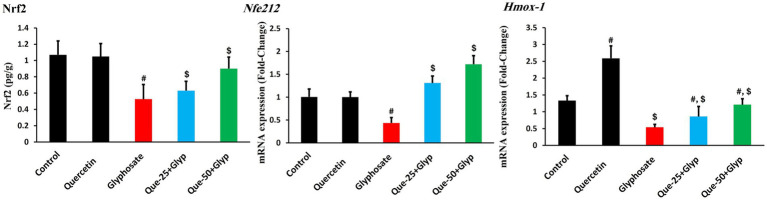
Effects of glyphosate and quercetin on serum renal function biomarkers and KIM-1. Rats were administered with glyphosate and/or quercetin at low dose or high dose for 21 days. Parameters measured include serum creatinine, urea, uric acid, and kidney tissue KIM-1 levels. Results were presented as mean± SD (*n* = 10). Statistical significance: ^#^ indicates significant difference compared to the control group (*p* < 0.05); ^$^ indicates significant difference compared to the glyphosate-treated group (*p* < 0.05).

Glyphosate exposure significantly impaired kidney function, as evidenced by pronounced elevation in serum creatinine, urea, and uric acid compared to controls (*p* < 0.05) (Figure 2). These results indicate a significant impairment of renal excretory function. KIM-1, a sensitive biomarker of proximal tubular damage, showed significant upregulation after glyphosate administration (*p* < 0.05, Figure 2), indicating that tubular epithelial injury is a primary event in glyphosate-induced nephrotoxicity. Quercetin treatment dose-dependently mitigated these impairments, with high-dose intervention (50 mg/kg/day) demonstrating superior normalization compared to low-dose treatment (25 mg/kg/day) and significantly reducing KIM-1 levels versus glyphosate-only exposure (*p* < 0.05), suggesting marked attenuation of tubular injury.

### 3.3 Oxidative stress markers and related gene expression

**Figure 3:**
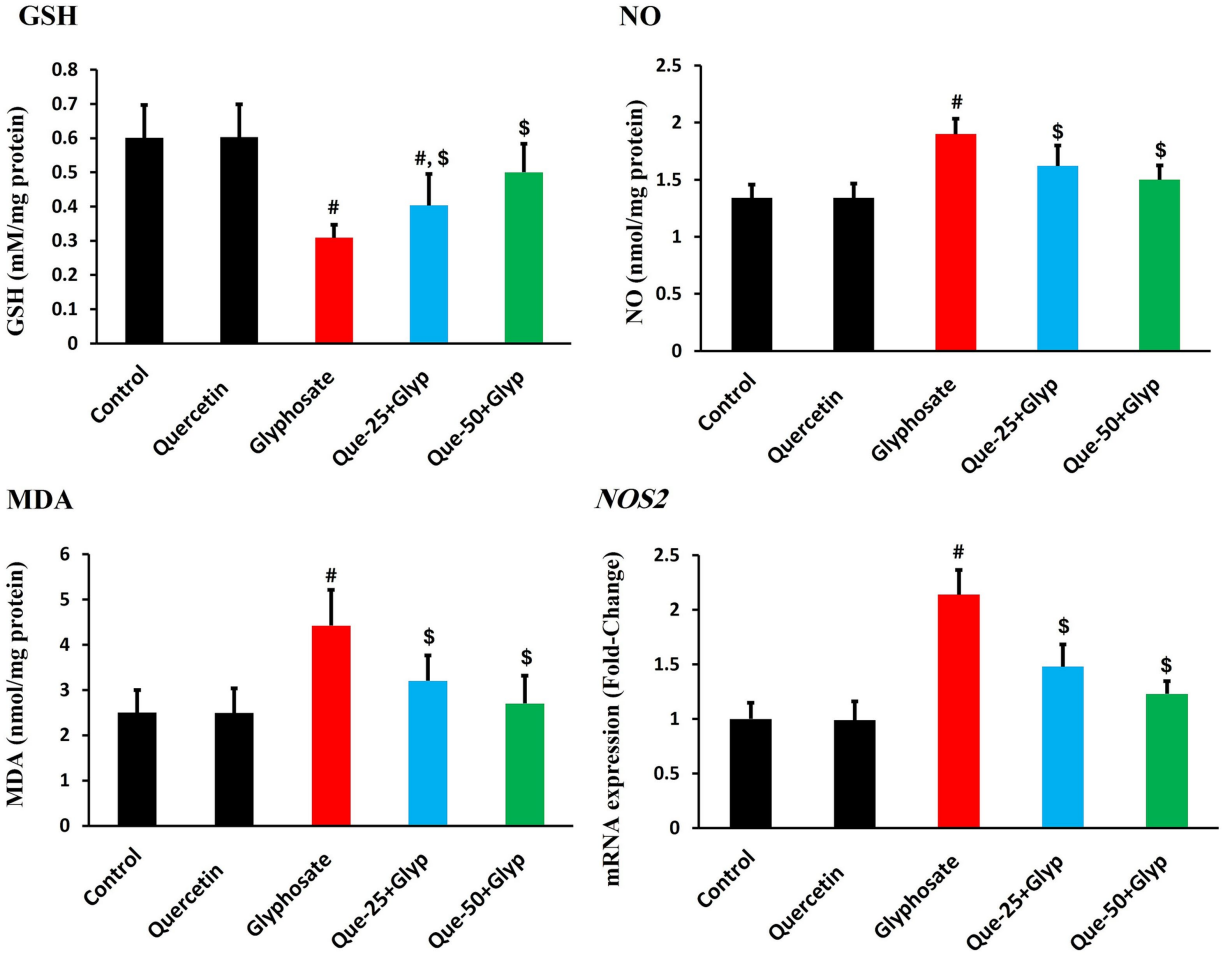
Effects of glyphosate and quercetin on oxidative stress parameters in renal tissue. Rats were administered with glyphosate and/or quercetin for 21 days. The figure shows levels of GSH, NO, MDA, and mRNA expression of inducible *NOS2*. Results were presented as mean± SD (*n* = 10). Statistical significance: ^#^ indicates significant difference compared to control (*p* < 0.05); ^$^ indicates significant difference compared to glyphosate group (*p* < 0.05).

Glyphosate exposure significantly disrupted redox homeostasis, as evidenced by profound alterations in oxidative stress parameters. GSH, a critical non-enzymatic antioxidant, was markedly depleted compared to control levels (*p* < 0.05), indicating severe compromise of cellular antioxidant capacity (Figure 3). Concurrently, MDA levels, indicative of lipid peroxidation, were dramatically elevated versus control (*p* < 0.05) (Figure 3), suggesting significant membrane integrity compromise.

Nitrosative stress parameters showed parallel dysregulation, with NO production substantially increased compared to control (*p* < 0.05, Figure 3). This elevation was accompanied by pronounced upregulation of inducible *NOS2* gene expression relative to control (*p* < 0.05), confirming enhanced nitrosative stress at both the metabolite and regulatory levels (Figure 3).

Quercetin intervention effectively attenuated these oxidative stress parameters in a dose-dependent pattern. High-dose quercetin (50 mg/kg) restored GSH content, approaching control values, while considerably reducing MDA (*p* < 0.05). Similarly, nitrosative stress markers were normalized, with NO levels and *NOS2* expression declining, comparable to control levels (*p* < 0.05). Low-dose quercetin (25 mg/kg) provided moderate protection across all parameters, though less effectively than the high-dose regimen (Figure 3).

### 3.4 Antioxidant parameters and related gene expression

**Figure 4:**
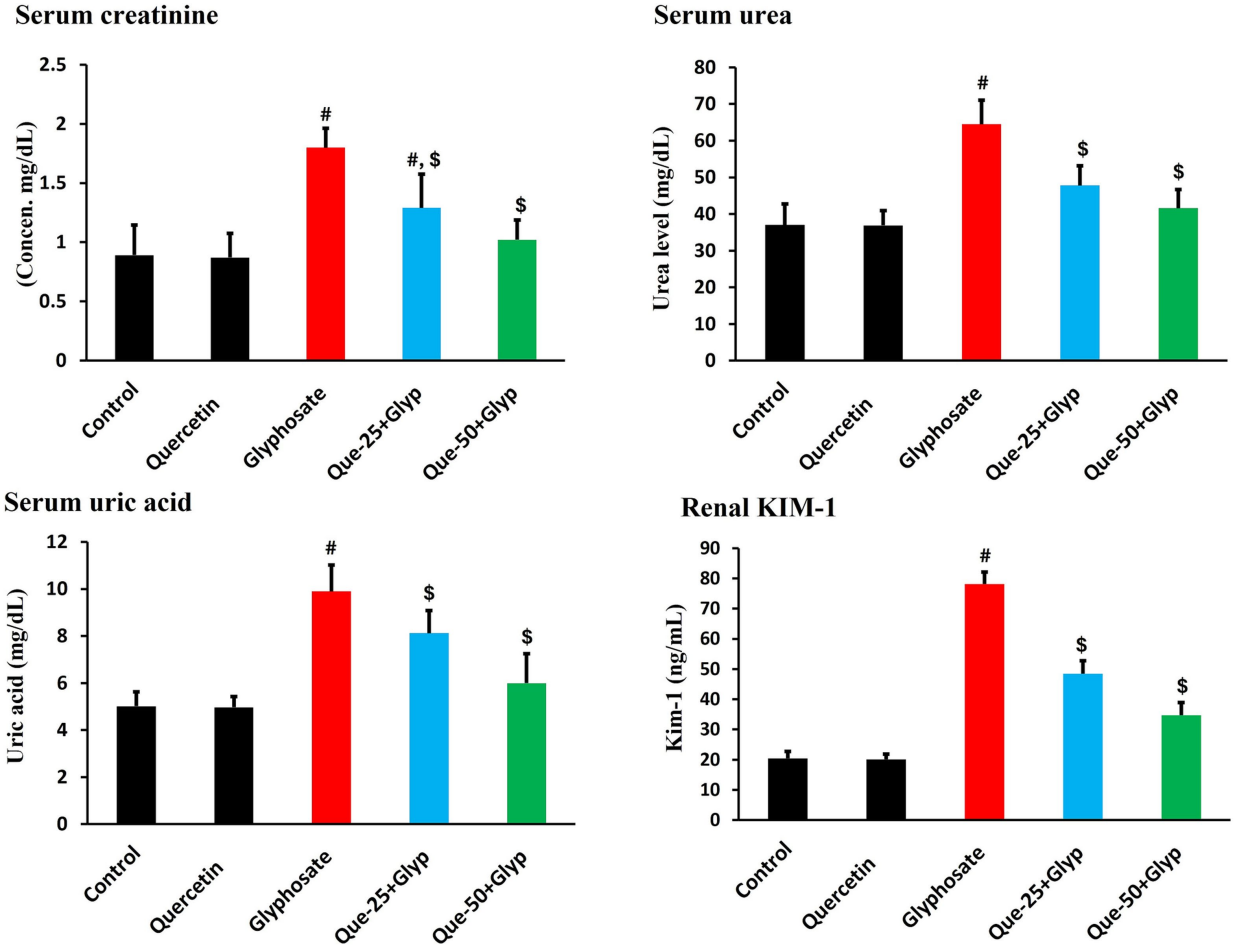
Effects of glyphosate and quercetin on antioxidant enzyme activities and their gene expression in renal tissue. Rats were administered with glyphosate and/or quercetin at low dose or high dose for 21 days. Left panels show enzyme activities of CAT, GPX, SOD, and GR. Right panels show corresponding mRNA expression levels (*Cat, Gpx-1,* and *Sod2*) presented as fold-change relative to control. Results were presented as mean± SD (*n* = 10). Statistical significance: ^#^ indicates significant difference compared to the control group (*p* < 0.05); ^$^ indicates significant difference compared to the glyphosate-treated group (*p* < 0.05).

Comprehensive assessment revealed marked impairment of antioxidant enzymes following glyphosate exposure. CAT activity was profoundly reduced compared to control (*p* < 0.05), with concurrent downregulation of *Cat* gene expression relative to control (Figure 4). Similarly, GPX activity substantially decreased (*p* < 0.05), with corresponding reduction in *Gpx-1* gene expression. SOD activity declined considerably (*p* < 0.05, Figure 4), with *Sod2* gene expression notably decreased. GR activity was likewise dramatically diminished compared to control (*p* < 0.05) (Figure 4).

Quercetin treatment dose-dependently restored these parameters. High-dose quercetin (50 mg/kg) effectively increased CAT, GPX, SOD, and GR activities compared to the glyphosate group (*p* < 0.05), with moderate improvement in the low-dose group (25 mg/kg). This protective effect extended to restoration of corresponding gene expression (*Cat, Gpx-1, Sod2*), with transcript levels approaching control values in the high-dose group (*p* < 0.05). Notably, quercetin monotherapy robustly upregulated antioxidant genes above baseline control levels (*p* < 0.05), suggesting potential priming effects that may enhance cellular resilience against subsequent oxidative challenges (Figure 4).

**Figure 5:**
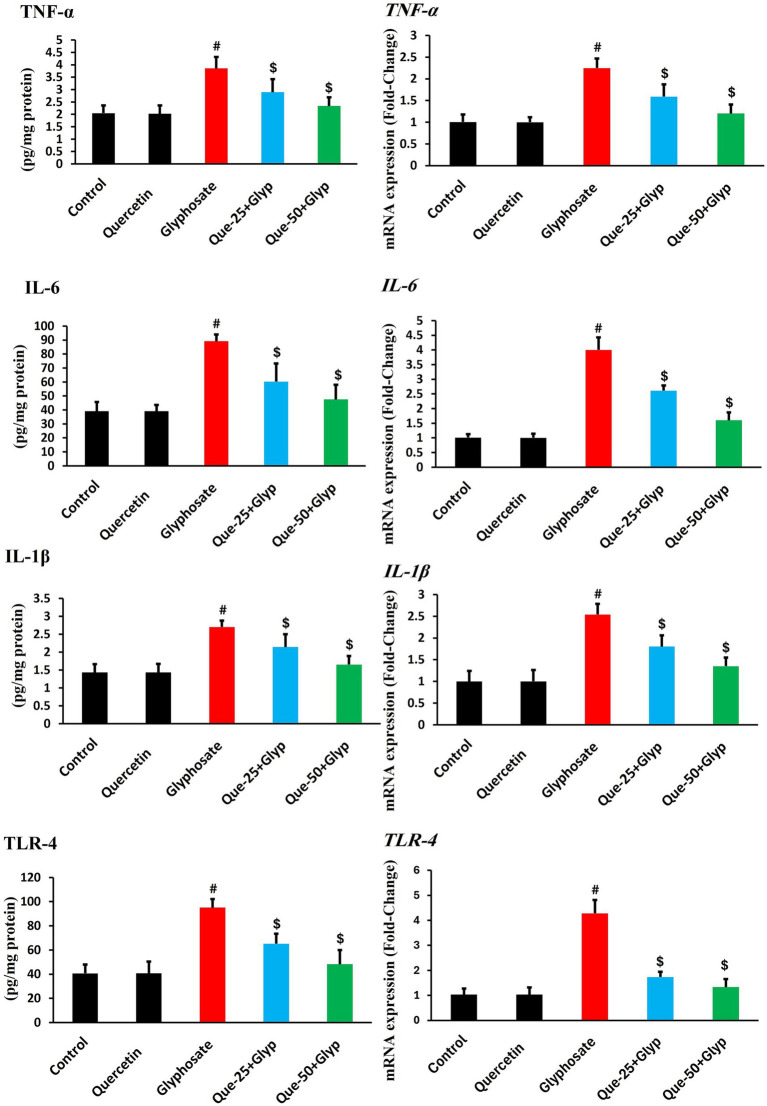
Effects of glyphosate and quercetin on Nrf2 protein levels and related gene expression in renal tissue. Rats were administered with glyphosate and/or quercetin for 21 days. The figure shows Nrf2 protein levels, and mRNA expression of *Nfe2l2* and *Hmox-1*, presented as fold-change relative to control. Results were presented as mean± SD (*n* = 10). Statistical significance: ^#^ indicates significant difference compared to control (*p* < 0.05); ^$^ indicates significant difference compared to glyphosate group (*p* < 0.05).

Nrf2, the master regulator of antioxidants, showed substantial reduction in protein levels compared to control (*p* < 0.05), accompanied by concurrent suppression of *Nfe2l2* gene expression, indicating inhibition of this master regulatory pathway at both transcriptional and post-transcriptional levels (Figure 5). Consequently, expression of direct target *Hmox-1* was pronouncedly downregulated (*p* < 0.05, Figure 5). High-dose quercetin appreciably ameliorated this dysregulation, increasing both Nrf2 protein levels and *Nfe2l2* gene expression compared to the glyphosate group (*p* < 0.05), with restoration of *Hmox-1* expression (Figure 5).

### 3.5 Inflammatory markers and related gene expression

**Figure 6:**
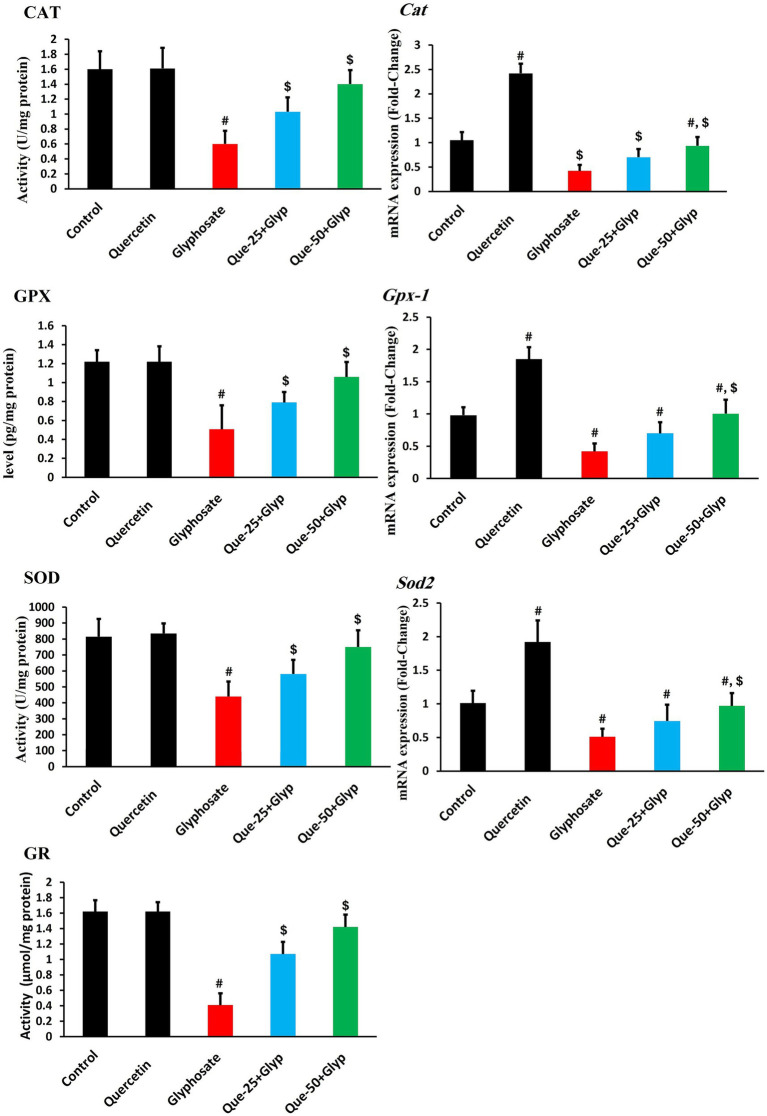
Effects of glyphosate and quercetin on inflammatory markers in renal tissue. Rats were administered with glyphosate and/or quercetin for 21 days. Left panels show protein levels of TNF-*α*, IL-6, IL-1*β*, and TLR-4. Right panels show corresponding mRNA expression presented as fold-change relative to control. Results were presented as mean± SD (*n* = 10). Statistical significance: ^#^ indicates significant difference compared to control (*p* < 0.05); ^$^ indicates significant difference compared to glyphosate group (*p* < 0.05).

Glyphosate exposure triggered pronounced inflammatory responses in renal tissue. Protein levels of TNF-*α*, IL-6, IL-1β, and TLR4 were markedly increased compared to control (*p* < 0.05), indicating activation of pro-inflammatory cytokine networks (Figure 6). Gene expression analysis revealed even more significant upregulation of these inflammatory markers (*p* < 0.05), with fold-changes exceeding those observed at the protein level, suggesting activation of common transcriptional regulatory mechanisms (Figure 6).

Quercetin treatment dose-dependently normalized both protein levels and gene expression of inflammatory markers. High-dose quercetin (50 mg/kg) showed superior anti-inflammatory effects compared to low-dose intervention (25 mg/kg), with significant reductions (*p* < 0.05) in both protein and transcript levels. Quercetin monotherapy did not significantly alter baseline inflammatory markers, indicating its effects were most pronounced in the context of pre-existing inflammatory activation (Figure 6).

### 3.6 Apoptotic markers and related gene expression

**Figure 7:**
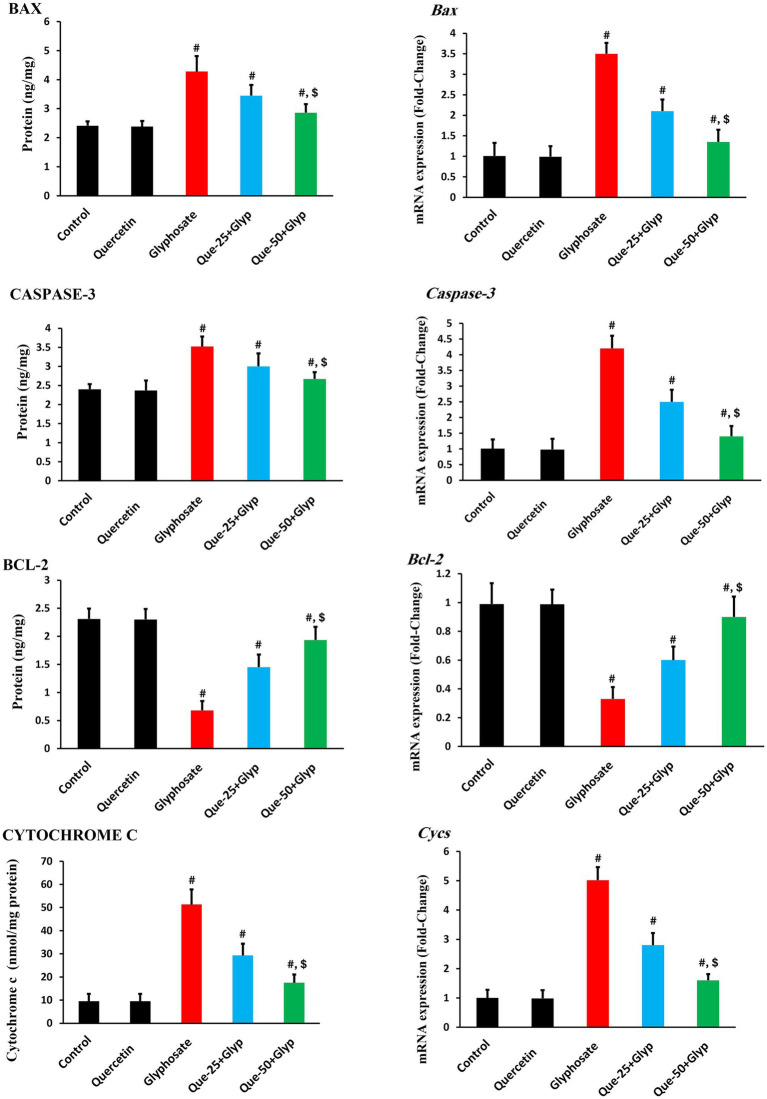
Effects of glyphosate and quercetin on apoptotic markers in renal tissue. Rats were administered with glyphosate and/or quercetin for 21 days. Left panels show protein levels of pro-apoptotic markers (BAX, CASPASE-3, CYTOCHROME C) and anti-apoptotic marker (BCL-2). Right panels show corresponding mRNA expression presented as fold-change relative to control. Results were presented as mean± SD (*n* = 10). Statistical significance: ^#^ indicates significant difference compared to control (*p* < 0.05); ^$^ indicates significant difference compared to glyphosate group (*p* < 0.05).

Glyphosate administration significantly dysregulated apoptotic pathways. Pro-apoptotic BAX, CASPASE-3, and CYTOCHROME C levels were markedly elevated (*p* < 0.05), indicating activation of the intrinsic mitochondrial apoptotic pathway. Anti-apoptotic BCL-2 protein levels were significantly decreased (*p* < 0.05) compared to control, dropping from approximately 2.3 ng/mg to 0.7 ng/mg, further confirming the shift toward pro-apoptotic signaling (Figure 7).

Gene expression analysis revealed upregulation of *Bax*, *caspase-3,* and *Cycs* genes (*p* < 0.05), consistent with protein findings. *Bcl-2* gene expression was significantly downregulated (*p* < 0.05) compared to control (Figure 7).

Quercetin treatment effectively normalized these apoptotic parameters dose-dependently. High-dose quercetin (50 mg/kg) demonstrated superior effects compared to low-dose treatment (25 mg/kg) (*p* < 0.05), restoring Bcl-2 protein levels to approximately 1.9 ng/mg and mRNA expression to 0.9-fold, while reducing pro-apoptotic markers and restoring the critical BAX/BCL-2 balance that regulates mitochondrial integrity and cellular survival (Figure 7).

### 3.7 Histopathological examination

**Figure 8:**
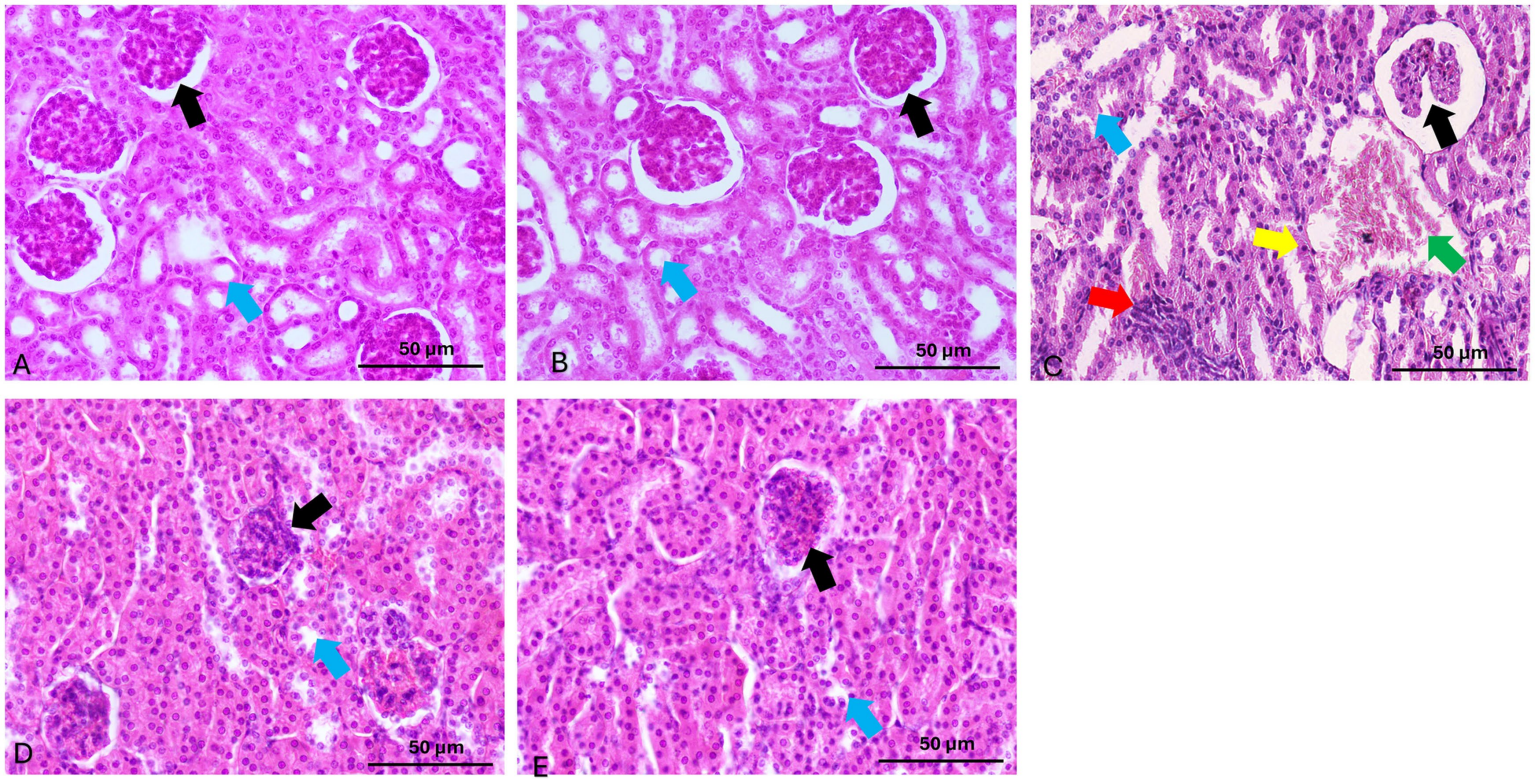
Representative photomicrographs of renal tissue sections stained with hematoxylin and eosin (H&E) from experimental groups. **(A,B)** Control and Quercetin groups show normal renal histology with intact glomeruli (black arrow) and tubules (blue arrow). **(C)** Glyphosate group reveals marked renal damage, including glomerular tuft atrophy with severe hemorrhage and congestion, cellular infiltration of the glomerular tuft (black arrow), leukocyte infiltration (red arrow), cloudy swelling (blue arrow), apoptosis (yellow arrow), and tubular dilation of the proximal tubule with hyaline casts (green arrow). **(D)** Que-25 + Glyp group shows glomeruli with reduced damage (black arrow) and preserved tubules (blue arrow). **(E)** Que-50 + Glyp demonstrates near-normal renal morphology with minimal histopathological changes in glomeruli (black arrow) and tubules (blue arrow). H&E stain, scale bar = 50 μm.

Histopathological assessment revealed distinct morphological differences among experimental groups. Kidney tissues collected from both the control and quercetin-treated groups exhibited normal renal architecture, with well-preserved glomeruli and intact tubular structures. In contrast, glyphosate-treated kidneys demonstrated marked renal damage, characterized by atrophy of the glomerular tuft with severe hemorrhage and congestion, inflammatory cellular infiltration of the glomerular tuft, interstitial leukocyte infiltration, epithelial cloudy swelling, extensive cellular apoptosis, and proximal tubular dilation with hyaline cast formation (Figure 8). These histopathological alterations confirm glyphosate-induced nephrotoxicity and correlate with the observed biochemical and molecular changes.

Quercetin treatment dose-dependently ameliorated these histopathological alterations. Low-dose quercetin (25 mg/kg) partially improved histological features, with moderate reduction in tubular degeneration and leukocytic infiltration. High-dose quercetin (50 mg/kg) showed marked improvement in renal architecture, with nearly normal glomeruli and minimal tubular damage. These morphological improvements corresponded with molecular findings, showing significant downregulation of pro-inflammatory and apoptotic genes and restoration of antioxidant genes in the high-dose quercetin group, suggesting comprehensive nephroprotection at both structural and molecular levels (Figure 8).

The semi-quantitative analysis revealed that glyphosate treatment significantly increased all renal damage parameters compared to control groups, with scores ranging from 2.0 to 3.0 across tubular dilatation, apoptotic cell abundance, glomerular atrophy, and leukocyte infiltration. Quercetin alone showed no significant histopathological changes compared to controls, confirming its safety profile. Both quercetin pretreatment groups demonstrated significant dose-dependent protective effects against glyphosate-induced nephrotoxicity, with the high-dose group (Que-50 + Glyp) showing superior protection, reducing damage scores to approximately 0.4–0.5 compared to 1.2–1.5 in the low-dose group (Que-25 + Glyp) as seen in Table 3.

**Table 3:** Semi-quantitative histopathological scoring of renal damage parameters in different experimental groups.

Group	Tubular dilatation	Apoptotic cell abundance	Glomerular atrophy	Leukocyte infiltration
Control	0.3 ± 0.48	0.2 ± 0.42	0.3 ± 0.48	0.3 ± 0.48
Quercetin	0.3 ± 0.67	0.2 ± 0.33	0.3 ± 0.67	0.3 ± 0.48
Glyphosate	3.1 ± 0.74 ^#, $^	2.3 ± 1.06 ^#, $^	2.3 ± 0.94 ^#, $^	2.5 ± 0.53 ^#, $^
Que-25 + Glyp	1.3 ± 0.67 ^#, $^	0.7 ± 0.62 ^#, $^	1.2 ± 0.78 ^#, $^	1.5 ± 0.52 ^#, $^
Que-50 + Glyp	0.4 ± 0.51 ^#, $^	0.3 ± 0.48 ^#, $^	0.4 ± 0.70 ^#, $^	0.5 ± 0.70 ^#, $^

Lesions were graded on a 0–4 scale for each component (0 = none, 1 = mild, 2 = moderate, 3 = severe, 4 = very severe). Data presented as mean ± SD (*n* = 10). Statistical significance: ^#^ indicates significant difference from control group (*p* < 0.05); ^$^ indicates significant difference from glyphosate group (*p* < 0.05).

## 4 Discussion

This study aimed to evaluate the nephrotoxic effects of glyphosate and investigate quercetin’s protective role against glyphosate-induced renal damage. Our findings revealed significant alterations at morphological, functional, biochemical, and molecular levels, confirming glyphosate’s nephrotoxicity and highlighting quercetin’s therapeutic potential.

The increased kidney weight after glyphosate administration represents a significant morphological manifestation of renal injury, likely reflecting inflammatory cell infiltration and cellular swelling, as reported by Karimi Jashni et al. (33). Quercetin dose-dependently normalized these parameters, consistent with its anti-inflammatory properties (34). Functional consequences of glyphosate exposure were evident through elevated renal biomarkers. Serum creatinine increased substantially, indicating impaired excretory function, with parallel rises in urea and uric acid levels, aligning with Nacano et al. (35).

Renal tubules, responsible for water and electrolyte reabsorption, were particularly vulnerable to toxic injury. Our study demonstrated that glyphosate exposure significantly damages proximal tubular epithelial cells, as evidenced by markedly elevated KIM-1 levels (*p* < 0.05) in exposed animals. KIM-1, a transmembrane glycoprotein specifically upregulated in injured proximal tubular cells, surpassed changes in conventional biomarkers, indicating the proximal tubular epithelium as the primary target of glyphosate toxicity. This finding aligns with Han et al. (36), who established KIM-1 as a sensitive biomarker for acute tubular damage. Our histopathological observations showing tubular epithelial degeneration provide morphological confirmation of this conclusion, suggesting that tubular injury precedes functional impairment. Reddy et al. (37) similarly reported that herbicide exposure primarily affects tubular structures before altering glomerular filtration. Importantly, quercetin treatment dose-dependently attenuated these tubular injury markers, demonstrating its specific protective effect against glyphosate-induced tubular damage.

Quercetin intervention attenuated these injury markers dose-dependently. Our findings provide substantial evidence that oxidative stress represents a central mechanism in glyphosate-induced renal injury. The disruption of the antioxidant defense system was evident through suppression of enzymatic antioxidants, with CAT, GPX, and SOD showing marked inhibition. This was accompanied by depletion of GSH content. Similar patterns have been documented by Zhou et al. (38), suggesting systematic disruption of cellular redox homeostasis. The consequences of this antioxidant defense collapse were manifested through elevation of oxidative damage markers, including MDA levels. Chang et al. (39) similarly reported that glyphosate-based formulations induce significant increases in oxidative stress biomarkers. The membrane peroxidation may compromise renal epithelial cell integrity, potentially explaining the glomerular and tubular damage observed histopathologically, as suggested by Romualdo et al. (40).

Concurrently, significant elevation in NO levels was observed following glyphosate exposure, accompanied by marked upregulation of *NOS2* gene expression. This indicates coordinated dysregulation of nitrosative stress at both metabolic and genetic regulatory levels, providing an additional mechanistic link between glyphosate exposure and renal injury. Quercetin treatment dose-dependently normalized both NO production and *NOS2* expression, suggesting that modulation of nitrosative stress represents an important mechanism contributing to quercetin’s nephroprotective effects.

Quercetin intervention effectively restored redox balance through multiple complementary mechanisms. The dose-dependent normalization of antioxidant enzyme activities (CAT, GPX, SOD) and GSH content suggests enhancement of endogenous antioxidant defense systems. Concurrently, the progressive reduction in oxidative damage markers (MDA and NO) indicates effective mitigation of free radical-mediated cellular injury. These findings align with reports by Alharbi et al. (41) and Zhang et al. (42), who documented quercetin’s capacity to restore antioxidant defenses and attenuate oxidative damage in various experimental models of oxidative stress-mediated tissue injury.

The molecular analysis of antioxidant gene expression revealed significant transcriptional dysregulation following glyphosate exposure, providing mechanistic insight into the observed enzymatic impairments. The coordinated downregulation of multiple antioxidant genes, including *Sod2, Cat, Gpx-1*, and *Hmox-1*, suggests suppressing a common transcriptional regulatory mechanism rather than gene-specific effects. The strong correlations between these expression patterns (*r*-values ranging from 0.82 to 0.88, all *p* < 0.05) further support their coordinated regulation through a shared pathway. Similar patterns of antioxidant gene suppression have been documented by Liu et al. (43) and Yengkokpam and Mazumder (44), who reported comparable transcriptional alterations in models of pesticide-induced oxidative stress.

The significant reduction in Nrf2 protein levels observed in our study provides mechanistic insight into the coordinated suppression of antioxidant genes. This protein reduction was accompanied by parallel downregulation of *Nfe2l2* gene expression, suggesting that glyphosate disrupts this pathway at multiple regulatory levels. As the master regulator of cellular antioxidant defense, Nrf2 controls numerous cytoprotective genes through binding to antioxidant response elements in their promoter regions. This dysregulation of the Nrf2-ARE pathway appears to be a central mechanism underlying the observed comprehensive impairment of antioxidant defense systems, creating a cellular environment highly susceptible to oxidative damage (45). Quercetin’s ability to restore both Nrf2 protein levels and gene expression likely represents a key mechanism behind its nephroprotective effects, as similarly reported by Wang et al. (46) in models of oxidative stress-induced renal injury.

Quercetin intervention effectively restored antioxidant gene expression in a dose-dependent manner, with high-dose quercetin (50 mg/kg) normalizing transcription of *Sod2, Cat, Gpx-1*, and *Hmox-1* to near-control levels. Notably, quercetin monotherapy induced substantial upregulation of these genes above baseline, with *Hmox-1* showing particularly pronounced induction. This transcriptional enhancement reveals quercetin’s capacity to activate antioxidant gene expression, as documented by Zhao et al. (47) and Kenan Kinaci et al. (48) across multiple experimental models.

The histopathological findings of leukocytic infiltration in glyphosate-exposed renal tissue were corroborated by molecular evidence of inflammatory pathway activation. Significant upregulation of TLR4 suggests recognition of glyphosate-induced damage-associated molecular patterns as inflammatory triggers, activating downstream signaling pathways. Similar TLR4-mediated inflammatory signaling has been reported by Wu et al. (49) in models of nephrotoxic acute kidney injury. Elevated expression of pro-inflammatory cytokines (TNF-*α*, IL-6, IL-1β) further confirms activation of inflammatory networks in response to glyphosate. These cytokines mediate local inflammatory responses and contribute to renal tissue injury through direct cytotoxic effects and promotion of oxidative stress. Inflammatory markers were markedly elevated in glyphosate-treated animals, highlighting inflammation as a key contributor to nephrotoxicity. Quercetin treatment effectively suppressed this inflammatory response in a dose-dependent manner, supporting its nephroprotective potential. The simultaneous improvement in both inflammatory and oxidative stress parameters suggests that these two pathways may be closely interlinked in the pathogenesis of glyphosate-induced renal damage. Similar anti-inflammatory effects of quercetin have been documented by Chen et al. (50). Our analysis revealed that inflammatory gene expression changes consistently exceeded and preceded protein alterations, suggesting significant post-transcriptional regulation during toxic stress. Quercetin treatment normalized both processes, with gene expression responding more rapidly than protein levels, indicating primary transcriptional regulatory effects. Similar regulatory patterns were reported Gaspard et al. (51) in xenobiotic-induced inflammatory responses.

Although cleaved CASPASE-3 is the definitive marker of irreversible apoptosis, this study relied on ELISA and qRT-PCR methods to evaluate CASPASE-3expression. While these approaches provide valuable insights into apoptotic activity, they do not differentiate between pro-CASPASE-3 and its cleaved active form. Future studies employing Western blot or immunohistochemistry would provide more direct confirmation of CASPASE-3 activation. Our findings establish the mitochondrial apoptotic pathway as a significant mechanism of cell death in glyphosate-induced nephrotoxicity. The observed upregulation of pro-apoptotic BAX coupled with downregulation of anti-apoptotic BCL-2 indicates disruption of the critical BAX/ BCL-2 balance governing mitochondrial membrane integrity. This mitochondrial destabilization is evidenced by significant CYTOCHROME C release, which is associated with the activation of the CASPASE cascade, as confirmed by elevated *caspase-3* expression and activity. Similar activation of the mitochondrial apoptotic pathway following glyphosate exposure has been reported by Lu et al. (52) and Gui et al. (11) in various cellular models.

The correlations between oxidative stress and apoptotic suggest that oxidative damage, particularly to cellular membranes, serves as a key contributor to the apoptotic cascade. The lipid peroxidation indicated by elevated MDA levels may compromise membrane integrity, particularly in organelles with high phospholipid content. Additionally, the impaired antioxidant defense, indicated by *Sod2* downregulation, likely exacerbates oxidative damage, further promoting cellular dysfunction and apoptotic signaling. Sule et al. (53) documented the relationship between oxidative stress and apoptotic activation in pesticide-induced cytotoxicity, and reported that ROS and RNS trigger mitochondrial apoptotic pathways.

Notably, we observed increased BCL-2 protein levels despite significant downregulation of *Bcl-2* gene expression, suggesting complex post-transcriptional regulatory mechanisms that may include enhanced protein stability or decreased degradation in response to toxic stress. This discordance highlights the importance of multi-level analysis for comprehensive understanding of cellular responses to toxicants.

Quercetin treatment effectively mitigated these apoptotic changes in a dose-dependent manner, normalizing the BAX/ BCL-2 ratio, reducing CYTOCHROME C release, and suppressing CASPASE-3 activation. This anti-apoptotic effect appears to result from multiple complementary mechanisms, including enhanced antioxidant protection as evidenced by restored antioxidant enzyme activities and reduced oxidative damage markers, which collectively help maintain cellular integrity and prevent progression to apoptotic cell death. These findings were consistent with reports by Chen et al. (54), who documented quercetin’s capacity to modulate apoptotic signaling in various models of toxicant-induced cell death. Specifically, Chen et al. (54) demonstrated that quercetin alleviates zearalenone-induced apoptosis and necroptosis in porcine renal epithelial cells by inhibiting the calcium-sensing receptor/calcium–calmodulin-dependent protein kinase II signaling pathway and by protecting the cells from oxidative damage caused by zearalenone exposure. Analysis of our findings suggests an integrated mechanistic model for glyphosate-induced nephrotoxicity centered on the interaction between inflammatory and oxidative pathways. Glyphosate exposure triggers inflammatory responses via *TLR-4* upregulation while suppressing antioxidant gene expression, creating a self-amplifying cycle that compromises mitochondrial integrity, activates apoptotic cascades, and results in tubular epithelial damage and functional impairment. Similar integrated mechanisms have been proposed by Ferrante et al. (55) in their review of glyphosate toxicity. Quercetin’s protective effects appear mediated through multiple complementary mechanisms: enhanced antioxidant gene expression (*Sod2, Cat, Gpx-1,* and *Hmox-1*) and restored enzyme activities, alongside attenuated inflammatory signaling through reduced expression of inflammatory mediators and *TLR-4.* These effects interrupt the inflammation-oxidative stress cycle, preserve mitochondrial integrity, and prevent apoptotic cell death as indicated by normalized CYTOCHROME C release, restored BCL-2 protein and gene expression levels, reduced BAX levels, and decreased CASPASE-3 activity. These multifaceted protective mechanisms align with findings by Song et al. (56). The dose-dependent nature of quercetin’s protective effects, with superior efficacy at 50 mg/kg, suggests potential therapeutic applications.

While this study provides significant insights into the protective effects of quercetin against glyphosate-induced nephrotoxicity, certain limitations should be noted. Firstly, the study was limited to a sub-acute exposure model in rats, which may not fully replicate chronic exposure scenarios in humans. Secondly, only male rats were used, and sex-specific differences were not explored. Thirdly, while multiple biomarkers and gene expressions were analyzed, more advanced molecular pathways (e.g., transcriptomics or proteomics) were not assessed. Additionally, while CASPASE-3 levels were assessed using ELISA and gene expression analysis, the absence of Western blot analysis to detect cleaved CASPASE-3 is a limitation that should be addressed in future research for confirming apoptotic pathway activation at the protein level. Future studies using chronic models, both sexes, and broader mechanistic approaches would help validate and expand these findings.

## 5 Conclusion

The present study provides comprehensive evidence that sub-acute glyphosate exposure induces significant nephrotoxicity in rats through a complex interplay of molecular mechanisms. Our findings demonstrate that glyphosate triggers renal damage via three interconnected pathways: (1) suppression of antioxidant defense systems and induction of oxidative stress, (2) activation of TLR-4-mediated inflammatory cascades, and (3) disruption of mitochondrial integrity leading to apoptotic cell death. These pathological processes collectively culminate in tubular epithelial damage, glomerular dysfunction, and impaired renal excretory capacity. Importantly, this study establishes quercetin as a potent nephroprotective agent against glyphosate-induced renal injury. Quercetin treatment, particularly at the higher dose (50 mg/kg), effectively normalized renal morphology and function, restored antioxidant enzyme activities and gene expression, attenuated inflammatory cytokine production, and inhibited the mitochondrial apoptotic pathway. The dose-dependent protective effects observed suggest a therapeutic threshold for optimal renoprotection against xenobiotic injury.

## Data Availability

The data presented in this study were available upon reasonable request from the corresponding author.
